# ASSOCIATIONS OF PLASMA GDF15 WITH PHYSICAL PERFORMANCE AND COGNITIVE FUNCTIONS IN OLDER ADULTS

**DOI:** 10.1093/geroni/igad104.0716

**Published:** 2023-12-21

**Authors:** Lingxiao He, Philipe Souto de Barreto, Juan Sánchez Sánchez, Yves Rolland, Sophie Guyonnet, Angelo Parini, Alexandre Lucas, Bruno Vellas

**Affiliations:** School of Public Health, Xiamen University, Toulouse, Midi-Pyrenees, France; CHU Toulouse: Centre Hospitalier Universitaire de Toulouse, Toulouse, Midi-Pyrenees, France; Gérontopôle de Toulouse, Toulouse, Midi-Pyrenees, France; Toulouse University Hospital (CHU Toulouse), Toulouse, Midi-Pyrenees, France; Gérontopôle de Toulouse, Toulouse, Midi-Pyrenees, France; Institute of Metabolic and Cardiovascular Diseases, Toulouse, Midi-Pyrenees, France; Institute of Metabolic and Cardiovascular Diseases, Toulouse, Midi-Pyrenees, France; Gérontopôle de Toulouse, Toulouse, Midi-Pyrenees, France

## Abstract

**Background:**

Growth differentiation factor 15 (GDF15) has been associated with several age-related disorders, but its associations with functional abilities in community-dwelling older adults are not well studied.

**Methods:**

The study was a secondary analysis on 1096 community-dwelling older adults (aged 69 to 94 years) recruited from the Multidomain Alzheimer’s Preventive Trial. Plasma GDF15 was measured one year after participants’ enrolment. Annual data of physical performance (grip strength and short physical performance battery [SPPB]) and global cognitive functions (mini-mental state examination [MMSE] and a composite cognitive score) were measured for four years. Adjusted mixed-effects linear models were performed for cross-sectional and longitudinal association analyses.

**Results:**

A higher GDF15 was cross-sectionally associated with a weaker grip strength (β = -1.1E-03, 95%CI [-2.0E-03, -1.5E-04]), a lower SPPB score (β = -3.1E-04, 95%CI [-5.4E-04, -9.0E-05]) and worse cognitive functions (β = -2.4E-04, 95%CI [-3.3E-04, -1.6E-04] for composite cognitive score; β = -4.0E-04, 95%CI [-6.4E-04, -1.6E-04] for MMSE). Participants with higher baseline GDF15 demonstrated greater longitudinal declines in SPPB (β = -1.0E-04, 95%CI [-1.7E-04, -2.0E-05]) and composite cognitive score (β = -2.0E-05, 95%CI [-4.0E-05, -3.6E-06]). The optimal initial GDF15 cutoff values for identifying participants with minimal clinically significant decline after one year were 2189 pg/mL for SPPB (AUC: 0.580) and 2330 pg/mL for composite cognitive score (AUC: 0.587).

**Conclusions:**

Plasma GDF15 is cross-sectionally and longitudinally associated with lower-limb physical performance and global cognitive function in older adults. Circulating GDF15 alone has limited capacity of discriminating older adults who will develop clinically significant functional declines.

